# Optimization of hydropower energy generation by 14 robust evolutionary algorithms

**DOI:** 10.1038/s41598-022-11915-0

**Published:** 2022-05-11

**Authors:** Mohammad Reza Sharifi, Saeid Akbarifard, Mohamad Reza Madadi, Kourosh Qaderi, Hossein Akbarifard

**Affiliations:** 1 Department of Hydrology and Water Resources, Faculty of Water & Environmental Engineering, Shahid Chamran University of Ahvaz, Ahvaz, Iran; 2 Ph. D. Graduate of Water Resources Engineering, Department of Hydrology and Water Resources, Faculty of Water & Environmental Engineering, Shahid Chamran University of Ahvaz, Ahvaz, Iran; 3 Department of Water Engineering, University of Jiroft, Jiroft, Iran; 4 Department of Water Engineering, Faculty of Agriculture, Shahid Bahonar University of Kerman, Kerman, Iran; 5 Department of Economics, Faculty of Management and Economics, Shahid Bahonar University of Kerman, Kerman, Iran

**Keywords:** Hydrology, Engineering, Mathematics and computing

## Abstract

The use of evolutionary algorithms (EAs) for solving complex engineering problems has been very promising, so the application of EAs for optimal operation of hydropower reservoirs can be of great help. Accordingly, this study investigates the capability of 14 recently-introduced robust EAs in optimization of energy generation from Karun-4 hydropower reservoir. The best algorithm is the one that produces the largest objective function (energy generation) and has the minimum standard deviation (SD), the minimum coefficient of variations (CV), and the shortest time of CPU usage. It was found that the best solution was achieved by the moth swarm algorithm (MSA), with the optimized energy generation of 19,311,535 MW which was 65.088% more than the actual energy generation (11,697,757). The values of objective function, SD and CV for MSA were 0.147, 0.0029 and 0.0192, respectively. The next ranks were devoted to search group algorithm (SGA), water cycle algorithm (WCA), symbiotic organism search algorithm (SOS), and coyote optimization algorithm (COA), respectively, which have increased the energy generation by more than 65%. Some of the utilized EAs, including grasshopper optimization algorithm (GOA), dragonfly algorithm (DA), antlion optimization algorithm (ALO), and whale optimization algorithm (WOA), failed to produce reasonable results. The overall results indicate the promising capability of some EAs for optimal operation of hydropower reservoirs.

## Introduction

Hydropower is the most important form of renewable energy in the world. It is often considered as the cheapest and very clean form of electricity^1^. In 2020, hydropower systems generated 16.8% (i.e. 4370 TWh) of the world’s total electricity generation and 70% of all renewable electricity^2^, and it is expected to remain the world’s primary source of renewable energy in 2024^3^. Regarding the increasing global demand for energy supply, the energy generation by hydropower reservoirs still needs to accelerate significantly. This necessitates the optimization of hydropower reservoirs operation. Optimization of reservoir operation is a complex engineering problem. This complexity arises from the stochastic nature of the system input, the nonlinearity of functions, the multiple constraints, the large number of decision variables, and other uncertainties. Therefore, solving this complex problem is out of the capability of classical methods, and needs more powerful techniques.

Evolutionary algorithms (EAs) demonstrated high performance in solving such complex engineering problems^4–6^. In water resources management, several EAs have been applied for solving reservoir optimization problems, including Genetic Programming, GP^7^, Genetic Algorithm, GA^8^, Particle Swarm Optimization, PSO^9^, Ant Colony Optimization, ACO^10^, Harmony Search, HS^11^, Imperialist Competitive Algorithm, ICA^12^, and comprehensive evolutionary algorithm, CEA^13^. In the last few years, Garousi-Nejad et al.^14^ reported the successful application of Firefly Algorithm (FA) in optimal operation of multi-purpose reservoirs. Chen et al.^15^, successfully employed an improved non-dominated sorting genetic algorithm-III (ENSGA-III) to optimize the reservoir operation during flood conditions. Ehteram et al.^16^ successfully applied Shark Algorithm (SA) to optimize the energy generation in a hydropower dam. Qaderi et al.^12^ documented the superiority of Water Cycle Algorithm (WCA) to HS and ICA in optimal operation of a multi reservoir system. Turgut et al.^17^ proposed the Master–Slave optimization algorithm for generating an optimal release policy of reservoir operation. Mohammadi et al.^18^ developed a new hybrid whale-genetic algorithm (HWGA) for optimal operation of 4- and 10-reservoir benchmark systems and documented its high efficiency. Feng et al.^19^ proposed the multi-strategy gravitational search algorithm (MGSA) for the optimal operation of cascade hydropower reservoirs.

Although the aforementioned EAs were widely applied in different engineering problems, there is no particular algorithm to gain the most appropriate solution for all optimization problems. Some algorithms may provide better solution for some particular problems but not for others. Therefore, it is necessary to evaluate the capability of each algorithm for each optimization problem.

Accordingly, this study investigates the capability of 14 robust EAs in the optimization of electricity generation from Karun-4 hydropower reservoir. These algorithms include coyote optimization algorithm, COA^20^, moth swarm algorithm, MSA^21^, grasshopper optimization algorithm, GOA^22^, dragonfly algorithm, DA^23^, whale optimization algorithm, WOA^24^, search group algorithm, SGA^25^, moth flame optimization algorithm, MFO^26^, ant lion optimizer algorithm, ALO^27^, symbiotic organisms search algorithm, SOS^28^, Krill Herd algorithm, KH^29^, water cycle algorithm, WCA^30^, gravitational search algorithm, GSA^31^, particle swarm optimization algorithm, PSO^32^ and genetic algorithm, GA^33^. The previous studies enumerated several superiorities of these EAs to many other methods including their simplicity, shorter run time, more exact results, good convergence speed and high convergence accuracy, strong robustness, parallel processing ability and low computational overhead. This study is the first application of most of the aforementioned algorithms in optimal operation of a real case hydropower reservoir.

## Materials and methods

As mentioned in the previous section, this study compares the capability of 14 robust algorithms in optimal operation of hydropower reservoirs. The remarkable performance of these EAs has made them the most successful algorithms among the more than two hundred algorithms in the literature. Here, a brief introduction to the utilized algorithms is presented. More mathematical explanations and details of these algorithms were previously presented by the cited references.

### COA algorithm

The coyote optimization algorithm (COA), which was inspired by the behavior of coyotes in nature, was first proposed by Pierezan and Coelho^20^. The first step in the COA algorithm is to initialize the decision variables and constraints as well as tuning the control parameters (number of packs, number of coyotes, scatter probability, association probability and eviction probability). The second step is to initialize randomly the social condition (adaptation) of each coyote, that is, random values are assigned respecting the constraints of the problem. The third step is to initialize the age of coyotes and to initialize the packs (coyotes are chosen randomly to compose the packs). The fourth step is to evaluate the fitness function of each coyote, that is, adaptation of the coyotes in the environment considering their social conditions. The fifth step is to define the alpha coyote (the leader of each pack). The sixth step is to calculate the cultural tendency of each pack. The cultural tendency of the pack is computed as the median social conditions of all coyotes from that specific pack. The seventh step is to generate a new social condition for each coyote. The new social condition is updated using the alpha and pack influence. The eighth step is to check the viability of the new positions and evaluate the fitness function of the new social conditions. The ninth step is to update the social condition of each coyote, that is, the coyote’s capacity decides if the new social condition is better than the older one to keep it. The tenth step is the birth of a coyote. The birth of a coyote is considered as a combination of the social conditions of two parents, randomly chosen, plus an environmental influence. The eleventh step is the death of a coyote. In order to keep the population size static, the COA syncs the coyote’s birth and death. The adaptation of the pup and adaptation of the group of coyotes is compared. If there is only one coyote with the worst adaptation, then it dies and the pup survives. If there are more than one coyote worst adapted, the eldest one dies and the pup survives. Otherwise, the pup dies. It is possible that two or more coyotes have similar age, in this case, the less adapted coyote is the one who dies. The twelfth step is the eviction of a coyote, which occurs with probability and it helps the COA to diversify the interaction between all the coyotes of the population. The thirteenth step consists on updating the coyotes' age and finally the social condition of the coyote that best adapted to the environment is selected and used as the global solution of the problem. These Steps (5–13) are repeated until a stop criterion is satisfied.

### MSA algorithm

Moth swarm algorithm (MSA), proposed by Mohamed et al.^21^, was inspired by the behavior of moths in the nature. The moths try to hide from predators during the day, while looking for the food resources at night with a celestial navigation technique. They fly in a straight line over a long distance by steer their locomotion in a steady angle relative to moonlight as the celestial far-distant point light. In the MSA, the possible solution is represented by position of light source, and the quality of this solution is considered as luminescence intensity of the light source. Three groups of moths (pathfinder, prospectors, and onlookers) are considered in the MSA. Pathfinders are capable to find the best position over the optimization space with First-In, Last-Out principle to guide the movement of the main swarm. Prospectors tend to wander into a random spiral path nearby the light sources, which have been marked by the pathfinders. Onlookers drift directly toward the best global solution (moonlight), which has been achieved by prospectors’ moths. In each iteration at MSA, each moth enters the problem to find the corresponding luminescence intensity of the light source. The best fitness in the population is considered as the position of pathfinder guiding for the next iteration. Thus, the second and third best groups are called prospectors and onlookers, respectively. The MSA algorithm is performed through three phases of initialization, reconnaissance, and transverse orientation. At the beginning of the flight, the position of each moths (initial solution) is randomly determined by a randomization function (initialization phase). Then, the type of each moth in the population is selected based on the fitness value (objective function). Thus, the best moth is considered as pathfinder (light sources) and the best and worst groups of moths are considered as prospectors and onlookers, respectively. During the prospecting process, the moths may be concentrated in some parts of the response space, led to entrapment in the local optima and reducing the quality of some moth populations. To prevent premature convergence and improve diversity in solutions, a part of the moth population is required to prospect the areas with less swarm. Pathfinders are responsible for this role. Thus, they update their position through interaction with each other and crossover operations and with the ability to fly long distances (known as lévy mutation) and prevent the stop in local optima (reconnaissance phase). The flight path of moths toward a light source can be described by cone-shaped logarithmic spirals. Accordingly, a set of paths located on the surface of the cone, with a fixed central angle, can describe the flight path of moths to the light source. A group of the moth with the highest luminescence intensities is selected as the prospectors. The number of prospectors should be reduced in each iteration (transverse orientation phase)^34,35^.

During the optimization process in the MSA, reducing the number of prospector moth increases the number of onlooker moth, leading to faster convergence to the global solution. Increased convergence velocity is in fact, due to the celestial navigation. An onlooker moth with the lowest luminescence can travel directly toward the best solution (moon). Hence, to control the recent movement, this step of the MSA algorithm is designed in such a way that onlookers are forced to search more effectively through focusing on important points of prospector. To this purpose, the onlookers are divided into two parts with Gaussian walk and associative learning mechanism. In the MSA, the type of each moth is alternately varied. Thus, each prospector that provides a better solution (greater luminescence than the light source) is promoted to the pathfinder. At the end of each step, the new light and moonlight sources will be available as possible solutions^36^.

### GOA algorithm

Grasshopper optimization algorithm (GOA), proposed by Mirjalili et al.^22^, simulates the swarming behavior of grasshoppers in the nature. In this algorithm, the position of the grasshoppers in the swarm represents a possible solution of a given optimization problem. The position of each grasshopper depends on three components of social interaction, gravity force and wind advection. In the swarm, a grasshopper might face three forces of attraction, repulsion, and neutral, depending on its location as compared to neighboring grasshoppers. A grasshopper will be in a neutral position (no force applied) when the distance is equal to a given value. As the grasshopper go further up to a certain value, it faces more attractive force and eventually for large distances, the magnitude of forces decreases. In this algorithm, the first step is started by generating random swarm as the initial solution to the problem. Then the cost of each grasshopper is determined by obtaining the value of the cost function. The process is continuous by absorbing the swarm via considered grasshoppers into their location to attract the grasshoppers to move into the considered grasshopper. Two main behaviors of the grasshoppers, long-range and abrupt movements of grasshoppers (exploration) and local movements to search for better food sources (exploitation), are considered.

### DA algorithm

Dragonfly algorithm (DA), proposed by Mirjalili^23^, is a recent metaheuristic algorithm inspired by the swarming behavior of dragonflies in nature. Dragonflies swarm for only two purposes: hunting and migration. The former is called static (feeding) swarm, and the latter is called dynamic (migratory) swarm. These two swarming behaviors are very similar to the two main phases of exploration and exploitation in optimization. In static swarm, dragonflies make small groups and fly back and forth over a small area to hunt other flying preys such as butterflies and mosquitoes (exploration phase). Local movements and abrupt changes in the flying path are the main characteristics of a static swarm. In dynamic swarms, however, a massive number of dragonflies make the swarm for migrating in one direction over long distances (exploitation phase). Considering these two behaviors, there are five main factors in position updating of individuals in swarms: separation, alignment, cohesion, attraction to food, distraction from enemy. The behavior of dragonflies is assumed to be the combination of these five corrective patterns. With these factors, different explorative and exploitative behaviors can be achieved during optimization. The dragonflies are required to change their weights adaptively for transiting from exploration to exploitation of the search space. It is also assumed that dragonflies tend to see more dragonflies to adjust flying path as optimization process progresses. In other word, the neighborhood area is increased as well whereby the swarm become one group at the final stage of optimization to converge to the global optimum. The food source and enemy are chosen from the best and worst solutions that the whole swarm is found so far. This causes convergence towards promising areas of the search space and divergence outward non-promising regions of the search space. To improve the randomness, stochastic behavior, and exploration of the dragonflies, they fly around the search space using a random walk (Levy flight) when there is no neighboring solution.

The DA algorithm starts optimization process by creating a set of random solutions for a given optimization problems. In fact, the position and step vectors of dragonflies are initialized by random values defined within the lower and upper bounds of the variables. In each iteration, the position and step of each dragonfly are updated. The position updating process is continued iteratively until the end criterion is satisfied.

### WOA algorithm

Whale optimization algorithm (WOA) is a swarm-based metaheuristic algorithm, proposed by Mirjalili and Lewis^24^. This algorithm is inspired by the bubble-net hunting strategy of humpback whales. Simply, bubble-net hunting behavior could be described such that humpback whales dive down approximation 12 m and then create the bubble in a spiral shape around the prey and then swim upward the surface following the bubbles. In WOA, the time-dependent location of a whale individual is measured by three operational processes of encircling prey, bubble-net attacking method (exploitation phase) and search for prey (exploration phase). In encircling phase, humpback whales discover the location of prey and encircle them. Since the position of the optimal design in the search space is not known a priori, the WOA algorithm assumes that the current best candidate solution is the target prey or is close to the optimum. After the best search agent is defined, the other search agents will hence try to update their positions towards the best search agent. In the next phase, a spiral mathematical formulation is created between the location of whale and prey to imitate the helix-shaped movement of humpback whales. In the search for prey phase, which is called the exploration phase, the whales use random search to discover their prey depending on the position of each other. Throughout the exploration phase, the location of a search agent is reorganized according to randomly selected search agent rather than the best search agent (exploitation phase). This procedure aids the WOA algorithm to perform the global search and overcome the local optimal problem.

### SGA algorithm

Search Group Algorithm (SGA), developed by Goncalves et al.^25^, is a robust metaheuristic algorithm. It is based on a good balance between the exploration and exploitation of the design domain. The basic idea in SGA is that in the first iterations of the optimization process, it tries to find promising regions on the domain (exploration), and as the iterations pass by, it refines the best design in each of these promising regions (exploitation). One of the principal parameters is the perturbation constant that controls this procedure. An important operator of SGA is the mutation that is employed to generate new individuals away from the ones of the current search group. However, the generation of new individuals is pursued only by a few members of the population, which represent the search group. In brief, SGA evolution is composed of five steps: initial population, initial search group selection, mutation of the search group, generation of the families and selection of the new search group. During the first step, an initial population is randomly generated. At the second step, all individuals of the population are evaluated and a search group is created by selecting a number of individuals from population. In order to perform this selection, SGA applies a standard tournament selection. In order to increase the global search ability of the proposed algorithm, the search group is mutated at each iteration. This mutation strategy consists in replacing mutated individuals from the search group by new individuals. The probability of a member of the search group to be replaced depends on its rank in it. In detail, an inverse tournament selection is employed since the winner of the tournament is the individual with the worst objective function value and consequently, it is replaced by a new one generated. The set comprised by each member of the search group and the individuals that it generated is denoted as family. Thus, once the search group is formed, each one of its members generates a family by the perturbation where perturbation constant controls the size of the perturbation and individuals. SGA is characterized by a reduction of the perturbation at each iteration of the evolution process. One of the features of SGA is that the better the quality of a member of the search group is, the more individuals it generates. At conclusion, the new search group is formed by the best member of each family. However, when the iteration number is higher than the global phase maximum number of iterations, the selection scheme is modified. The new search group is formed by the best individuals among all the families. This phase is called local because the algorithm will tend to exploit the region of the current best design.

### MFA algorithm

Moth–flame optimization algorithm (MFO) was proposed by Mirjalili^26^, based on the behavior of moth in nature. It imitates the moths’ movement technique in the night, called transverse orientation for navigation. In this method, a moth flies by maintaining a fixed angle with respect to the moon, a very effective mechanism for travelling long distances in a straight path. MFO combines a population-based algorithm and local search strategy to yield an algorithm capable of global exploration and local exploitation. In the algorithm, it is assumed that the candidate solutions are moths and the problem’s variables are the position of moths in the space. Therefore, the moths can fly in the space with changing their position vectors. The steps of the MFO optimization starts by initializing the positions of moths within the solution space. Each moth updates it’s position with respect to a flame based on a spiral equation. The control parameters are linearly decreased over iterations to emphasize exploitation. In each iteration, the flames list is updated and then sorted based on the fitness values of flames. Consequently, the moths update their positions with respect to their corresponding flames. To increase the chance of reaching to the global best solution, the number of flames is decreased with respect to the iteration number. Thus, a given moth updates its position using only one of the flames. This process continues until the termination criteria are met.

### ALO algorithm

The ant lion optimization algorithm (ALO) mimics the hunting mechanism of ant lions in nature. It was first introduced by Mirjalili^27^. Ant lions are in the family of *Myrmeleontidae* and belong to the order of Neuroptera. The life cycle of ant lions includes two main phases, larva and adult, which takes 2 to 3 years. The ant lions’ life cycle mostly occurs in larvae and adulthood has only 3 to 5 weeks. The larvae of ant lions are also known as doodlebugs, which have a predatory habit. Adult ant lions can fly and maybe are mistakenly identified as dragonflies or damselflies. The name of “ant lions” best describes their unique hunting behavior and their favorite prey which is ants. The larvae of some ant lion’s species dig cone-shaped pits with different sizes and wait at the bottom of the pits for ants or other insects to slip on the loose sands and fall in. When an insect is in a trap, the ant lion will try to catch it while the trapped insect will try to escape. The ant lion intelligently tries to slide the prey into the bottom of the pit by throwing sands toward the edge of the pit. After catching the prey, the ant lion pulls it under the soil and consumes it. After feeding is completed, the antlion flicks the leftovers of the prey out of the pit and prepares the pit for next hunting. It should be noted that the size of the ant lion’s trap depends on the level of antlion hunger and the shape of the moon. Ant lions dig larger pits when they become hungry and also when the moon is full. For larger pits, the chance of successful hunting increases.

In the ALO algorithm, ants are search agents and move over the decision space, and ant lions are allowed to hunt them and become fitter. In the ALO, the first positions of ant lions and ants are initialized randomly and their fitness functions are calculated. Then, the elite antlion is determined. In each iteration for each ant, one ant lion is selected by the roulette wheel operator and its position is updated with the aid of two random walk around the roulette selected antlion and elite. The new positions of ants are evaluated by calculating their fitness functions and comparing with those of ant lions. If an ant becomes fitter than its corresponding ant lion, its position is considered as a new position for the ant lion in the next iteration. Also, the elite will be updated if the best antlion achieved in the current iteration becomes fitter than the elite. These steps are repeated until the end of iterations.

### SOS algorithm

The symbiotic organism search algorithm (SOS), proposed by Cheng and Prayogo^28^, is based on the cooperative behavior among organisms in nature. Organisms in the real world do not live alone because they are interdependent on other species for sustenance and survival. The interdependency between two discrete species is known as symbiotic. In this context, mutualism, commensalism, and parasitism are the most common symbiotic relations found in the nature. Interdependency between two different species that results in mutual benefit is called mutualism. A relationship between two different species that offers benefits to only one of them (without the affecting other) is called commensalism. Finally, a relationship between two different species that offers benefits to one and cause harm to the other is called parasitism.

The SOS begins with an initial population called ecosystem. In the initial ecosystem, a group of organisms (decision variables) is produced randomly in the search space. In the first step of the search process lifecycle, three organisms, *P*_*best*_, *P*_*i*_ and *P*_*j*_, are selected from the ecosystem. In the selection process, both *P*_*i*_ and *P*_*best*_ organisms are determined by the deterministic method. *P*_*best*_ is the solution candidate with the highest fitness value in the ecosystem and selected by the greedy method. *P*_*i*_ represents the solution candidates selected from the ecosystem in order of their index by the ordinal selection method. *P*_*j*_ is randomly selected from the ecosystem. The second step of the search process lifecycle of the SOS algorithm consists of three phases: mutualism, commensalism and parasitism. In these three stages, the same *P*_*best*_ and *P*_*i*_ organisms are used. In other words, the *P*_*best*_ and *P*_*i*_ organisms in the mutualism phase and those used in commensalism, and parasitism stages are the same. *P*_*j*_ is determined randomly in three stages. The overall process of this algorithm is as follows:

$${\text{Initialization}}\, \to \,{\text{Repeat}}\, \to \,{\text{Mutualism phase}}\, \to \,{\text{Commensalism phase}}\, \to \,{\text{Parasitism phase}}\, \to \,{\text{Termination criterion is met}}.$$

### KH algorithm

The Krill Herd (KH) algorithm, introduced by Gandomi and Alavi^29^, is inspired by the simulation of the herding behavior of the small crustaceans (krill) who spend their life under the water. One of the basic properties of this type of crustaceans is the ability to form large swarms/herds to avoid predators. Like other metaheuristic algorithms, KH has special fitness function to solve global optimization problems based on the food location and swarm density. In KH algorithm, each krill tries to be in the highest density area and at the same time it keeps looking to most places containing food. Increasing density and finding food are used as objectives to lead the krill to the global optima at the end. In other word, the objective function for the movement of krill is measured by the shortest distance of each individual krill from food and highest density of the herd. In the movement process, each krill moves toward the best solution based on three operational processes: movement induced by other krill individuals, foraging activity, and random diffusion. The KH algorithm is being referred to as a powerful search technique because it contains both exploration and exploitation strategies based on foraging movement and the motion induced by other individuals respectively. One of the remarkable advantages of the KH algorithm is that the KH does not need the derivative information because it uses a stochastic search rather than a gradient search.

### WCA algorithm

The water cycle algorithm (WCA) is a robust metaheuristic algorithm inspired by water cycle process in nature^30^. It mimics the flow of rivers and streams toward the sea. A river or a stream is formed whenever water moves downhill from one place to another. On their downhill journey and eventually ending up to a sea, water is collected from rain and other streams. Also, water in rivers and lakes is evaporated while plants transpires. The evaporated water is carried into the atmosphere to generate clouds which then condenses in the colder atmosphere, releasing the water back to the earth in the form of rain or precipitation. This process is called the water cycle. In the real world, as snow melts and rain falls, most of water enters the aquifer (groundwater). The ground water may be discharged into a stream (marsh or lake), which will be next evaporated and bring more clouds and thus more rain as this cycle counties.

WCA algorithm begins with an initial population called the raindrops. The best individual (best raindrop) is chosen as a sea. Then, a number of good raindrops are chosen as rivers and the rest of the raindrops are considered as streams which flow to the rivers or may flow directly into the sea. Depending on their magnitude of flow, each river absorbs water from the streams. In fact, the amount of water in a stream entering rivers and/or sea varies from other streams. In addition, rivers flow to the sea which is the most downhill location. For the exploitation phase of the WCA, new positions for streams and rivers are considered. If the solution given by a stream is more optimal than that of its connecting river, the positions of the river and stream are exchanged (i.e., the stream becomes a river and the river becomes a stream). A similar exchange can be performed for a river and the sea. The evaporation process operator is also introduced to avoid premature convergence to local optima. After evaporation, the raining process is applied and new streams are formed in different locations. Indeed, the evaporation operator is responsible for the exploration phase in the WCA. Uniform random search is used to specify the new locations of the newly formed streams. Control parameters prevents additional searches or search intensity near the sea.

### GSA algorithm

The gravitational search algorithm (GSA) is a powerful evolutionary algorithm which relies upon the Newtonian’s law of motion and law of gravity of masses to describe the interaction between the agents. It was first intended by Rashedi et al.^31^. The gravitational forces between two agents is directly proportional to the product of their masses and inversely proportional to their distance squared. Furthermore, a gravitational constant exist which is changing during the course of time. Two terms of active mass (which indicates the strength of a gravitational field due to its mass), and passive mass (which represents the strengths of an object interaction with gravitational field) are defined. Besides, based on the Newtonian’s law of motion, the acceleration, is directly proportional to the net force acting on the particle, and is inversely proportional to the mass of the particle. Based on law of gravity, there is an attracting gravity force among all particles of the universe. Among the particles, the effect of bigger and the closer particles are higher. On the contrary, an increase in the distance between two particles will decrease the gravity force between them. GSA considers agents as objects of different masses and they move to each other due to gravity force. Their performance is measured by their masses and lighter objects move towards objects with heavier masses. Each object is specified by four parameters which are position, inertial mass, active gravitational mass, and passive gravitational mass. The gravitational and inertial masses are evaluated using a fitness function and they control the velocity of an object in the specified dimension. The positions of objects in specified dimensions are updated with each iteration. The termination of the algorithm is defined by a fixed number of iterations and the recorded best fitness at final iteration becomes the global fitness for the particular problem. The position of the mass at specified dimensions of the corresponding object becomes the global solution of the problem. Generally, the steps of GSA include search space identification, randomized initialization, fitness evaluation of agents, updating the control parameters, calculation of the total force in different directions, calculation of acceleration and velocity, updating agents’ position, repeating these steps until the stop criteria is reached, and finally the end.

### PSO algorithm

The particle swarm optimization algorithm (PSO), proposed by Kennedy and Eberhart^32^, is a metaheuristic algorithm inspired from swarm behavior of flocks of birds or schools of fish in nature. These swarms follow a cooperative way to find food, and each member in the swarms keeps changing the search pattern according to the learning experiences of its own and other members. While searching for food, the birds are either scattered or go together before they locate the place where they can find the food. While the birds are searching for food from one place to another, there is always a bird that can smell the food very well, that is, the bird is perceptible of the place where the food can be found, having the better food resource information. Because they are continuously exchange information about the food place, the birds will eventually flock to the place where better food can be found.

In the PSO algorithm, solution swam is equal to the bird swarm, the birds’ moving from one place to another is equal to the development of the solution swarm, good information is equal to the most optimist solution, and the food resource is equal to the most optimist solution during the whole course. The position of each particle in the swarm is affected both by the most optimist position during its movement and the position of the most optimist particle in its surrounding. In other word, the movement of each particle is identified in two phases of exploration (global search) and exploitation (local search). In the exploration phase, particle fly across the whole search space to find a limited region containing the global optimum. After the region containing the global optimum has been found, the exploitation phase is started. The position of each particle is corrected by taking small movements in the neighborhood of the global optimum. By adopting the correct sequence of these two phases, it is possible to lead particles towards the global optimum^37^.

### Genetic algorithm

The Genetic Algorithm (GA), proposed by Holland^33^, is one of the most popular EAs that is inspired by Charles Darwin’s theory of natural evolution. This algorithm reflects the process of natural selection where the fittest individuals are selected for reproduction in order to produce offspring of the next generation. GA algorithm starts with an initial set of random solutions, called population. Each solution in the population is called a chromosome, which represents a point in the search space. The chromosomes evolve through successive iterations, called generations. During each generation, the chromosomes are evaluated using some measures of fitness. The fitter the chromosomes, the higher the probabilities of being selected to perform the genetic operations, including crossover and mutation. In the crossover phase, the GA attempts to exchange portions of two parents, that is, two chromosomes in the population to generate an offspring. The crossover operation speeds up the process to reach better solutions. In the mutation phase, the mutation operation maintains the diversity in the population to avoid being trapped in a local optimum. A new generation is formed by selecting some parents and some offspring according to their fitness values, and by rejecting others to keep the population size constant. After the predetermined number of generations is performed, the algorithm converges to the best chromosome, which hopefully represents the optimal solution or may be a near-optimal solution of the problem^37^.

## Case study: Karun-4 hydropower reservoir system

**Figure 1:**
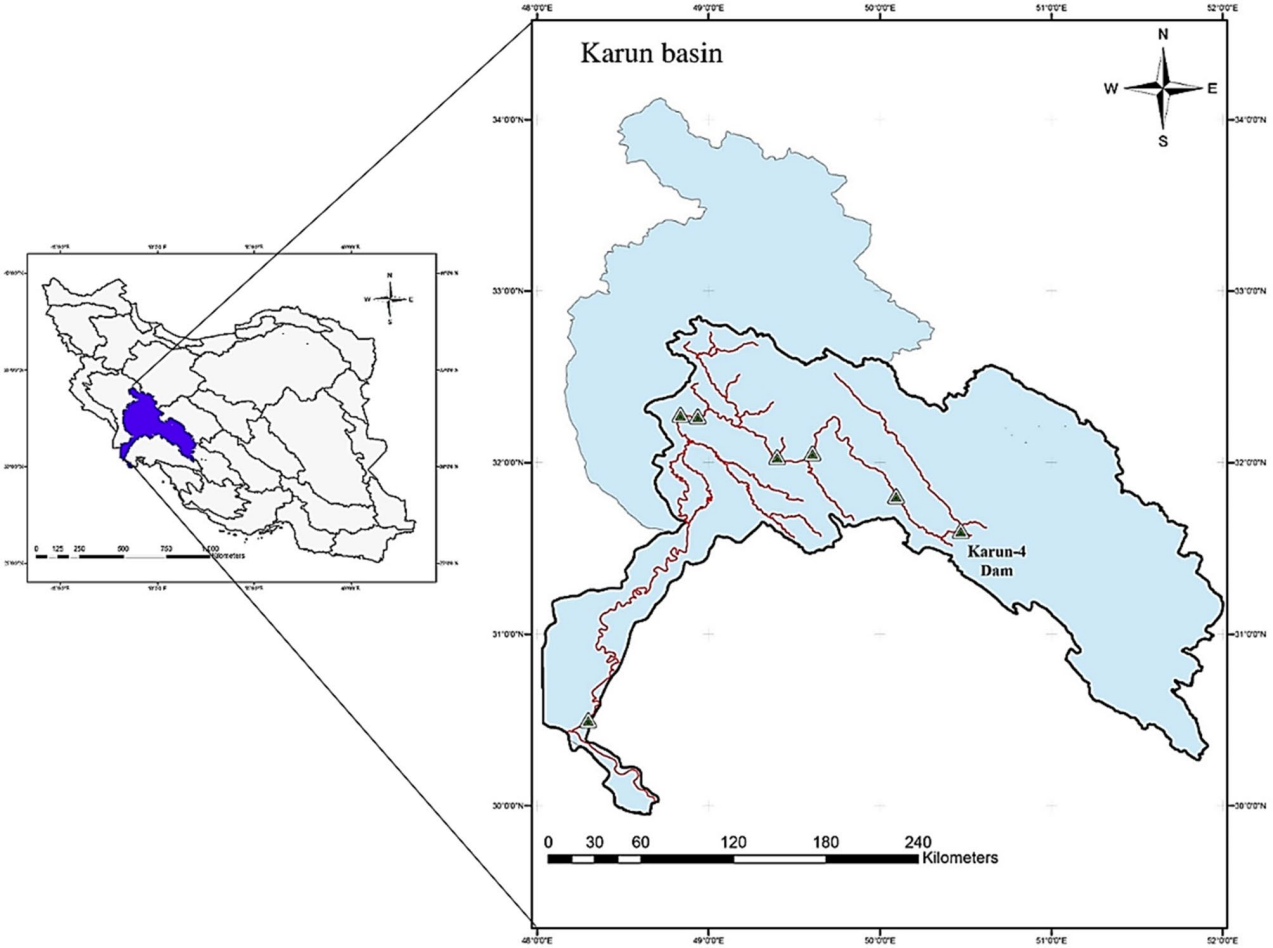
Location of the Karun-4 hydropower dam in the Karun basin (southwest of Iran).

To compare the performance of utilized algorithms, the optimal operation of Karun-4 hydropower reservoir in terms of electricity generation was studied. Karun-4 hydropower dam is the tallest dam in Iran, constructed on the Karun River at the southwest of Iran (Fig. 1). This dam has 4 hydropower plants with a design capacity of 1000 MW which supplies the demands of region. More characteristics of this dam was presented in Table 1.

**Table 1:** Characteristics of the Karun-4 hydropower dam.

Parameters	Unit	Value
North latitude	Degree	31° 35′
East longitude	Degree	50° 24′
Minimum reservoir storages	MCM	1405
Maximum reservoir storages	MCM	2279
Power plant capacity (PPC)	MW	1000
Annual potential energy production	MWh	2107
Efficiency	Percent (%)	80

## Modeling procedure

For maximization of electricity generation by Karun-4 hydropower reservoir, an optimization model with monthly time step was structured during a 10-years period, 2010 to 2019. The objective function and constraints regarding the developed model are as follows:

1 $$MinF=\sum_{t=1}^{T}\left(1-\frac{{P}_{t}}{PPC}\right)+Penalty,$$

2 $$P_{t} = g \times e_{t} \times {\text{ }}\left( {\frac{{RP_{t} }}{{PF}}/Mul_{t} } \right) \times \left( {\overline{{H_{t} }} - TW_{t} } \right)/1000,$$

3 $$\overline{{H_{t} }} = (H_{t} + H_{t + 1} )/2,$$

4 $$H_{t} = a_{0} + a_{1} \cdot S_{t} + a_{2} \cdot S_{t}^{2} + a_{3} \cdot S_{t}^{3} ,$$

5 $$TW_{t} = b_{0} + b_{1} \cdot {\text{Re}}_{t}^{Power} + b_{2} \cdot ({\text{Re}}_{t}^{Power} )^{2} + b_{3} \cdot ({\text{Re}}_{t}^{Power} )^{3} ,$$

6 $$RPS_{t} = {\text{Re}}_{t}^{Power} - RP_{t} ,$$

7 $$0 \le P_{t} \le PPC,$$

8 $$S_{t + 1} = S_{t} + Q_{t} - {\text{Re}}_{t}^{Power} - Sp_{t} - Loss_{t} ,$$

9 $$Loss_{t} = (Ev_{t} - R_{t} ) \times \overline{{A_{t} }} /1000,$$

10 $$\overline{{A_{t} }} = (A_{t} + A_{t + 1} )/2,$$

11 $$A_{t} = c_{0} + c_{1} \cdot S_{t} + c_{2} \cdot S_{t}^{2} + c_{3} \cdot S_{t}^{3} ,$$

12 $$S_{\min } \le S_{t} \le S_{\max } ,$$

where *PPC* is total power plant capacity (MW), $P_{t}$ is the electricity generated by the power plant (MW), and *T* is a total month of hydropower operation of the Karun-4 dam. In addition, $e_{t}$ is the efficiency of the power plant, *g* is the gravitational acceleration, $RP_{t}$ is the release of water through the power plant to generate power in month *t* (MCM), *PF* is the plant factor, $Mul_{t}$ is the factor of conversion from million cubic meters to cubic meters per second during month *t*, $TW_{t}$ is reservoir tail-water level which is assumed constant for all periods during month *t* (m), $\overline{{H_{t} }}$ is the average reservoir water level at the month *t* (m), $H_{t}$ and $H_{t + 1}$ are reservoir water level at the beginning and end of month t (m), $RPS_{t}$ is the overflow volume of hydropower outlet in month t (MCM), ${\text{Re}}_{t}^{Power}$ is the release of water through the power plant in month *t* (MCM) (${\text{Re}}_{t}^{Power}$ is a decision variable in the hydropower optimization problem, the aim is to obtain the best value of ${\text{Re}}_{t}^{Power}$ in each month, so that the hydropower generation is maximized), $Q_{t}$ is the reservoir inflow in month *t* (MCM),$S_{t}$ is the reservoir storage in month *t* (MCM), $Sp_{t}$ is the overflow volume from the reservoir during month *t* (MCM), $Loss_{t}$ is the loss from reservoir in month *t* (MCM), $R_{t}$ is the depth of precipitation on reservoir in month *t* (m), $Ev_{t}$ is the depth of evaporation from reservoir in month t (m), $A_{t}$ and $A_{t + 1}$ are area of the reservoir lake at the beginning and end of month t (Km^2^), $S_{\max }$ is the maximum storage capacity (MCM), $S_{\min }$ is the minimum storage (MCM), and $a_{i}$, $b_{i}$ and $c_{i}$ are the coefficients of the Storage-Area-Depth relationships for the Karun-4 reservoir.

The reservoir storage should not be less than the minimum storage (*S*_*min*_) and more than the maximum storage (*S*_*max*_). For this purpose, a penalty function is defined in Eq. (13). Since the algorithm tries to minimize the objective function, the penalty function is added if the constraint of reservoirs storage is not met.

13 $$Penalty=\left\{\begin{array}{l}\sum_{t=1}^{T}{({S}_{t}-{S}_{min})}^{2}\; if \,{S}_{t}<{S}_{min}\\ \sum_{t=1}^{T}{({S}_{t}-{S}_{maxi})}^{2}\; if \,{S}_{t}>{S}_{max}\\ 0 if {S}_{t}\ge {S}_{min} and {S}_{t}\le {S}_{max}\end{array}\right..$$

The search space of the Karun-4 hydropower optimization problem including the type of variables and their ranges was presented in Table 2.

**Table 2:** Variables of the Karun-4 hydropower optimization problem.

Variable	Type of variable	Lower bound	Upper bound	Unit
Hydropower release (*Re*^*Power*^)	Decision variable	5	1240	MCM
Reservoir storage (*S*)	State variable	1405	2279	MCM
Reservoir inflow (*Q*)	State variable	80	1820	MCM
Reservoir area (*A*)	State variable	22.05	30.1	km^2^
Reservoir water level (*H*)	State variable	996	1032	m
Tail-water level (*TW*)	State variable	840	863	m
Electricity generation (*P*)	State variable	5	1000	MW
Plant factor (*PF*)	State variable	20	25	%

All the algorithms were coded in MATLAB 2016a based on PC with i7 CPU 1.8 GHz/16 GB RAM/2 TB HDD. The values of algorithms setting parameters for operation of Karun-4 hydropower dam were presented in Table 3. This table provides the best values of algorithms’ parameters based on the sensitivity analysis. In order to achieve reliable results, 10 independent runs of each algorithm were compared. Furthermore, to have a fair comparison, the population size and the number of iterations were considered the same.

**Table 3:** Values of algorithms parameters for Karun-4 hydropower reservoir operation.

MSA	Iterations	Number of variables	Number of search agents	Number of pathfinders	–
	1000	106	100	20	–
GA	Iterations	Number of variables	Number of genes	Mutation rate	Crossover rate
	1000	106	100	0.01	0.8
PSO	Iterations	Number of variables	Population size	C1	C_2_
	1000	106	100	1.49	1.49
ALO	Iterations	Number of variables	Number of search agents	–	–
	1000	106	100	–	–
COA	Iterations	Number of variables	Number of packs	Number of coyotes	–
	1000	106	100	50	–
DA	Iterations	Number of variables	Number of search agents	–	–
	1000	106	100	–	–
GOA	Iterations	Number of variables	Number of search agents	–	–
	1000	106	100	–	–
GSA	Iterations	Number of variables	Number of solutions	G0	Alpha
	1000	106	100	1000	1
KH	Iterations	Number of variables	Number of krills	Crossover flag	-
	1000	106	100	Yes	–
MFO	Iterations	Number of variables	Number of search agents	–	–
	1000	106	100	–	–
SGA	Iterations	Number of variables	Population size	Global iterations ratio	Search group ratio
	1000	106	100	0.3	0.1
SOS	Iterations	Number of variables	Ecosystem population size	BF1	BF2
	1000	106	100	1 or 2	1 or 2
WCA	Iterations	Number of variables	Number of rain drops	Number of rivers and sea	dmax
	1000	106	100	50	1.5
WOA	Iterations	Number of variables	Number of search agents	–	–
	1000	106	100	–	–

## Results and discussion

**Figure 2:**
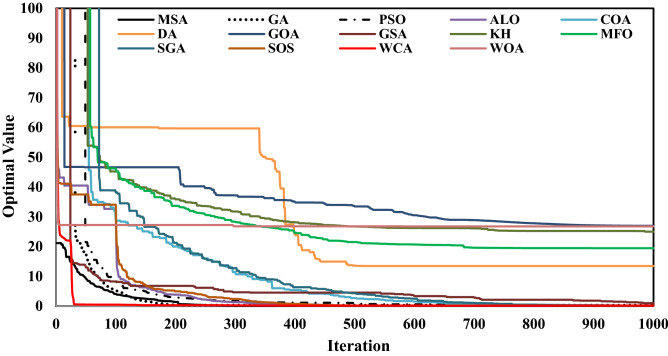
The convergence rate of utilized algorithms in optimization of Karun-4 hydropower reservoir.

In evaluation of EAs, it is important to understand how fast EAs converge to the optimum solution, or their convergence rates. Figure 2 demonstrates the convergence rate of utilized algorithms. As shown, the WCA and MSA had the fastest convergence rate, they could approach the values close to the optimal by the least number of iterations (less than 200 iterations). Also, GA, PSO, ALO and SOS demonstrated satisfactory results in terms of reach to the optimal value. But the GOA, WOA and KH demonstrated the slowest convergence rate, they could not reach well to the optimal value even after more than 1000 iterations.

Table 4 demonstrates the values of objective function for 10 runs of utilized algorithms. As seen, the best values of the objective function belong to the MSA (0.1470) with the least SD (= 0.0029) and CV (= 0.0192). In addition, it has the shortest CPU time (19.70 s), which indicates its highest efficiency. The powerful operators in MSA algorithm, by balancing the capabilities of exploration and exploitation, have been able to perform more efficiently than the other algorithms in the large-scale and complex problem of Karun-4 hydropower reservoir.

**Table 4:** The values of objective function for 10 runs of utilized algorithms.

Number of runs	GA	PSO	GSA	WCA	KH	SOS	ALO	MFO	SGA	WOA	DA	GOA	MSA	COA
1	1.6918	0.1584	2.1114	0.1554	25.0249	0.1486	0.5534	22.6256	0.1473	38.5649	29.8341	29.0066	0.1559	0.3245
2	1.4352	1.0708	1.2747	0.1509	27.4642	0.1499	2.3789	25.2252	0.1520	45.7274	20.8790	50.6324	0.1473	0.5233
3	1.9616	0.2499	1.4319	0.1513	33.7400	0.1477	0.1530	28.0716	0.1502	41.6850	35.8575	33.9242	0.1470	0.2858
4	1.4702	0.5463	1.9794	0.1561	30.5381	0.1493	0.8962	19.4558	0.1491	42.0414	21.3502	27.5000	0.1486	0.2727
5	0.3762	0.2756	1.9238	0.1532	29.3031	0.1591	0.8693	26.5767	0.1485	35.7049	46.2809	26.9784	0.1508	0.2672
6	0.6623	0.1704	1.9045	0.2350	28.3926	0.4741	2.3380	23.2866	0.1505	26.7870	13.4671	43.1339	0.1472	0.1780
7	1.3717	0.2570	1.0652	0.1562	30.0798	0.1473	0.1507	29.1343	0.1528	42.1593	71.4737	36.3977	0.1506	0.1494
8	0.9225	0.1591	0.9835	0.2025	29.0006	0.1473	1.4238	23.4442	0.1529	35.2602	23.4197	32.3821	0.1470	0.1740
9	0.5495	0.732	1.3332	0.1527	30.0887	0.1518	1.5399	22.3391	0.2618	28.9443	14.3605	29.1482	0.1473	0.4782
10	0.3026	0.1823	1.1816	0.1563	28.2805	0.1502	0.7395	23.2970	0.1503	49.3276	32.4972	29.0753	0.1471	0.2056
Best	0.3026	0.1584	0.9835	0.1509	25.0249	0.1473	0.1507	19.4558	0.1473	26.7870	13.4671	26.9784	**0.1470**	0.1494
Worst	1.9616	1.0708	2.1114	0.2350	33.7400	0.4741	2.3789	29.1343	0.2618	49.3276	71.4737	50.6324	**0.1559**	0.5233
Average	1.0744	0.3802	1.5189	0.1670	29.1912	0.1825	1.1043	24.3456	0.1615	38.6202	30.9420	33.8179	**0.1489**	0.2859
SD	0.5864	0.3078	0.4194	0.0284	2.2597	0.1024	0.8019	2.9153	0.0352	7.0885	17.4358	7.6911	**0.0029**	0.1267
CV	0.5458	0.8096	0.2761	0.1701	0.0774	0.5612	0.7261	0.1197	0.2183	0.1835	0.5635	0.2274	**0.0192**	0.4433
Best CPU time (s)	37.16	28.88	120.47	43.17	935.89	47.30	425.59	224.95	42.08	108.22	64.84	258.92	**19.70**	50.61
Overall rank	6	6	8	3	10	4	12	9	2	11	13	14	1	5

Significant values are in bold.

Based on the values of parameters of objective function, standard deviation, coefficient of variations and the CPU time, a ranking system was utilized for a better comparison of the algorithms performance^38^. In this ranking system, the rank of each algorithm in terms of each parameter was computed and then the overall rank was determined regarding the summation of these individual ranks. Based on the ranking system, the first rank belongs to MSA followed by SGA, WCA and SOS respectively. The MSA had the shortest CPU run time among all, representing its remarkable performance. On the other hand, the algorithms of GOA, DA, ALO and WOA failed to produce reasonable results, so they had the lowest ranks among the utilized algorithms.

The real energy generation versus the optimized values by the utilized algorithm were presented in Table 5. As seen, in the real condition, the total hydropower generation over 106 months of reservoir operation was 11,697,757 MW, averagely 64,988 MW per month. The use of EAs could improve the energy generation up to 65% and even more. In terms of energy generation, the highest performance belongs to MSA with 19,311,535 MW (65.0875% increase), and the lowest performance belongs to GOA with 14,398,451 MW (23.0873% increase) energy generation. Although all the utilized EAs increased the energy generation, the highest energy generation was respectively attributed to the MSA, SGA, SOS, COW, ALO, WCA, and PSO with more than 65% increase in energy generation. It is found from the table that, the optimization of hydropower reservoir operation is of great importance, specially, in a situation where energy consumption is increasing day by day in the world.

**Table 5:** The optimized values of energy generation vs. the current condition.

	Optimized energy (MW)	Increase compared to the current condition (%)
MSA	19,311,535	65.0875
SGA	19,311,485	65.0871
SOS	19,311,476	65.0870
COA	19,311,094	65.0837
ALO	19,310,867	65.0818
WCA	19,310,824	65.0814
PSO	19,309,490	65.0700
GA	19,283,532	64.8481
GSA	19,160,514	63.7965
DA	16,885,330	44.3467
MFO	15,790,536	34.9877
KH	14,764,656	26.2178
WOA	14,447,075	23.5029
GOA	14,398,451	23.0873
Current condition	11,697,757	–

**Figure 3:**
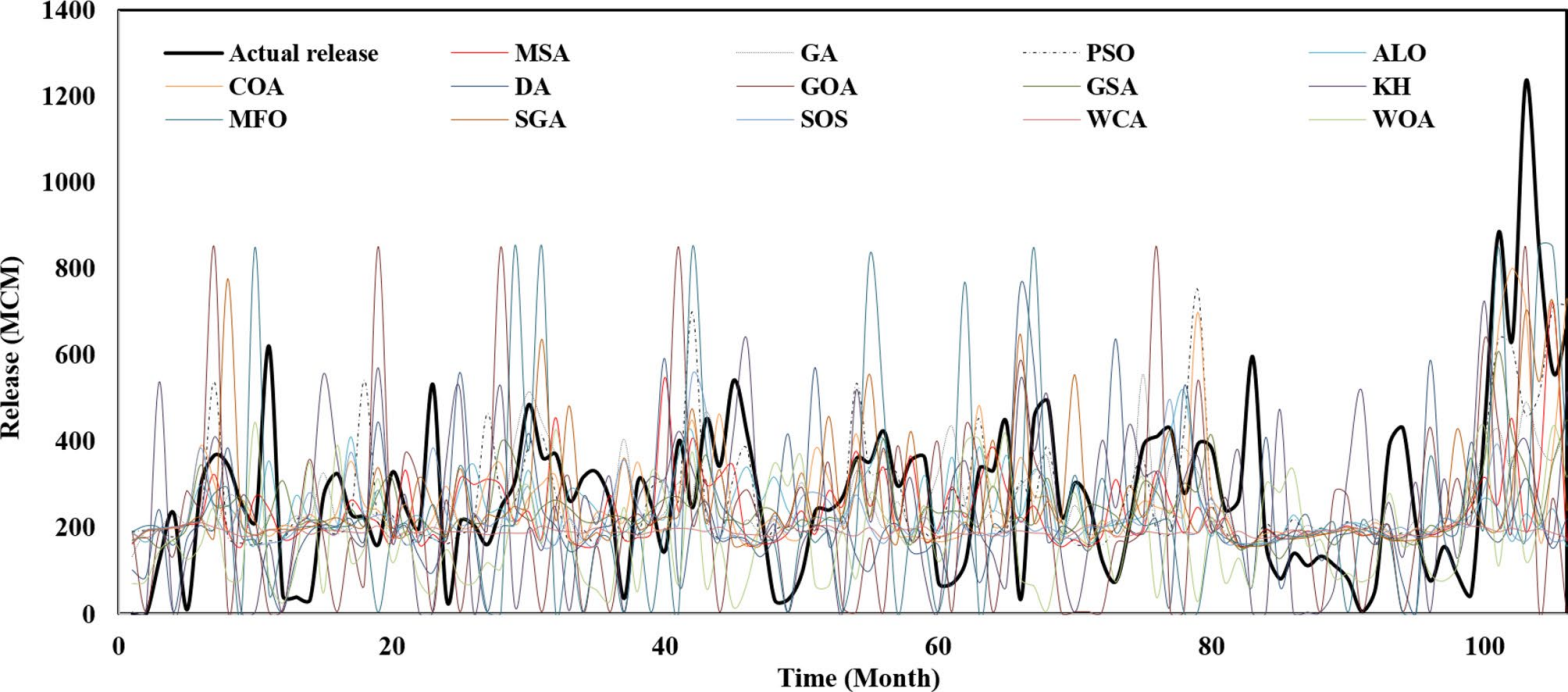
The actual release pattern of Karun-4 hydropower reservoir vs. predicted values by the algorithms.

**Figure 4:**
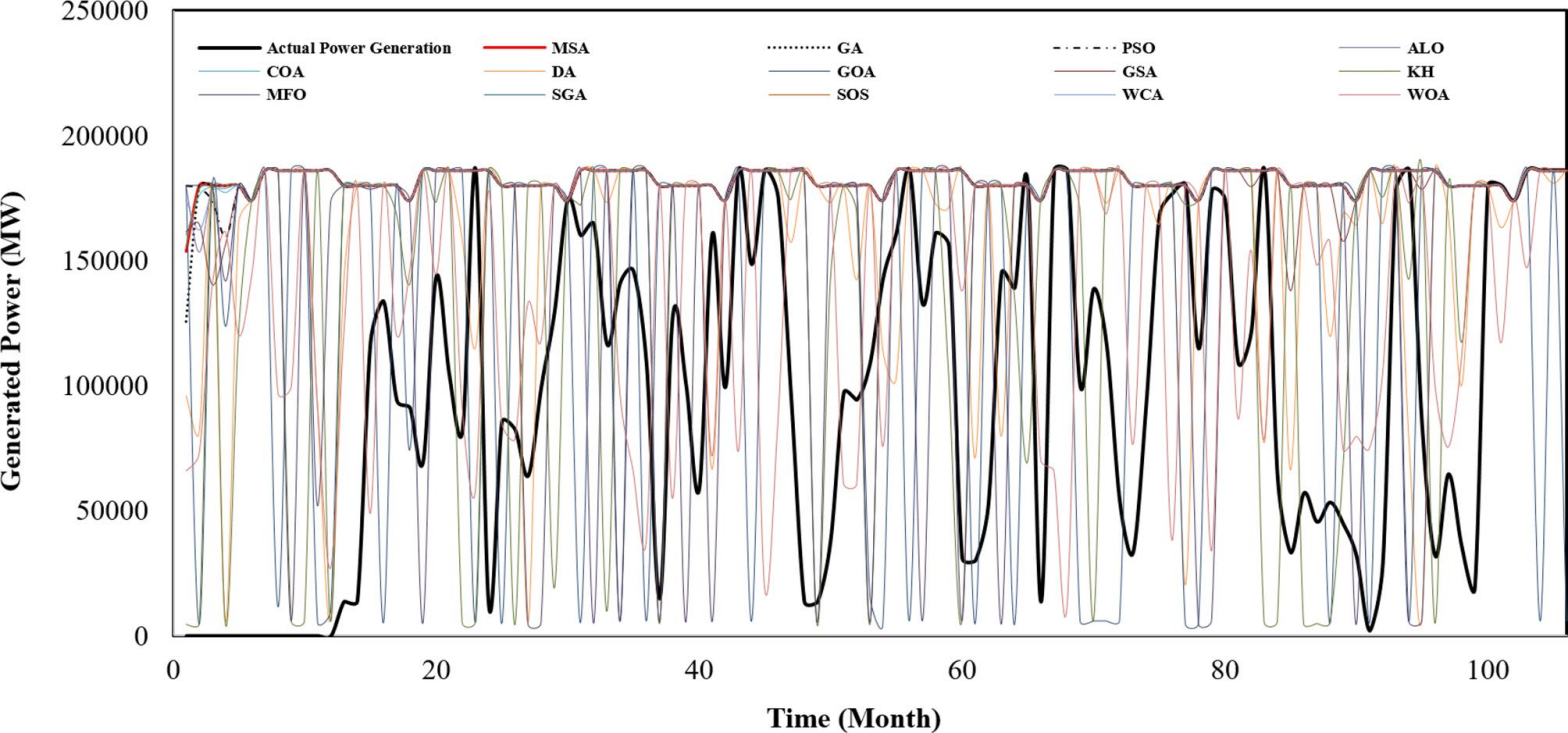
The actual power generation patterns of Karun-4 hydropower reservoir vs. predicted values by the algorithms.

Figure 3 represents the actual release pattern of Karun-4 hydropower reservoir versus the predicted values by the utilized algorithms. This can be employed by water policymakers as a guide (rule curve) to schedule the water release from the Karun-4 dam in a way that the most generation of hydropower energy is obtained. The real energy generation pattern of Karun-4 hydropower reservoir versus the predicted values by the algorithms has been presented in Fig. 4. It is clearly observed that the energy generation in real condition is significantly less than the optimized condition in almost all the months of the study period. As seen, the operating policies obtained by MSA, SGA and SOS have resulted the maximum energy generation with a more appropriate release pattern, so that the system does not face shortages.

**Figure 5:**
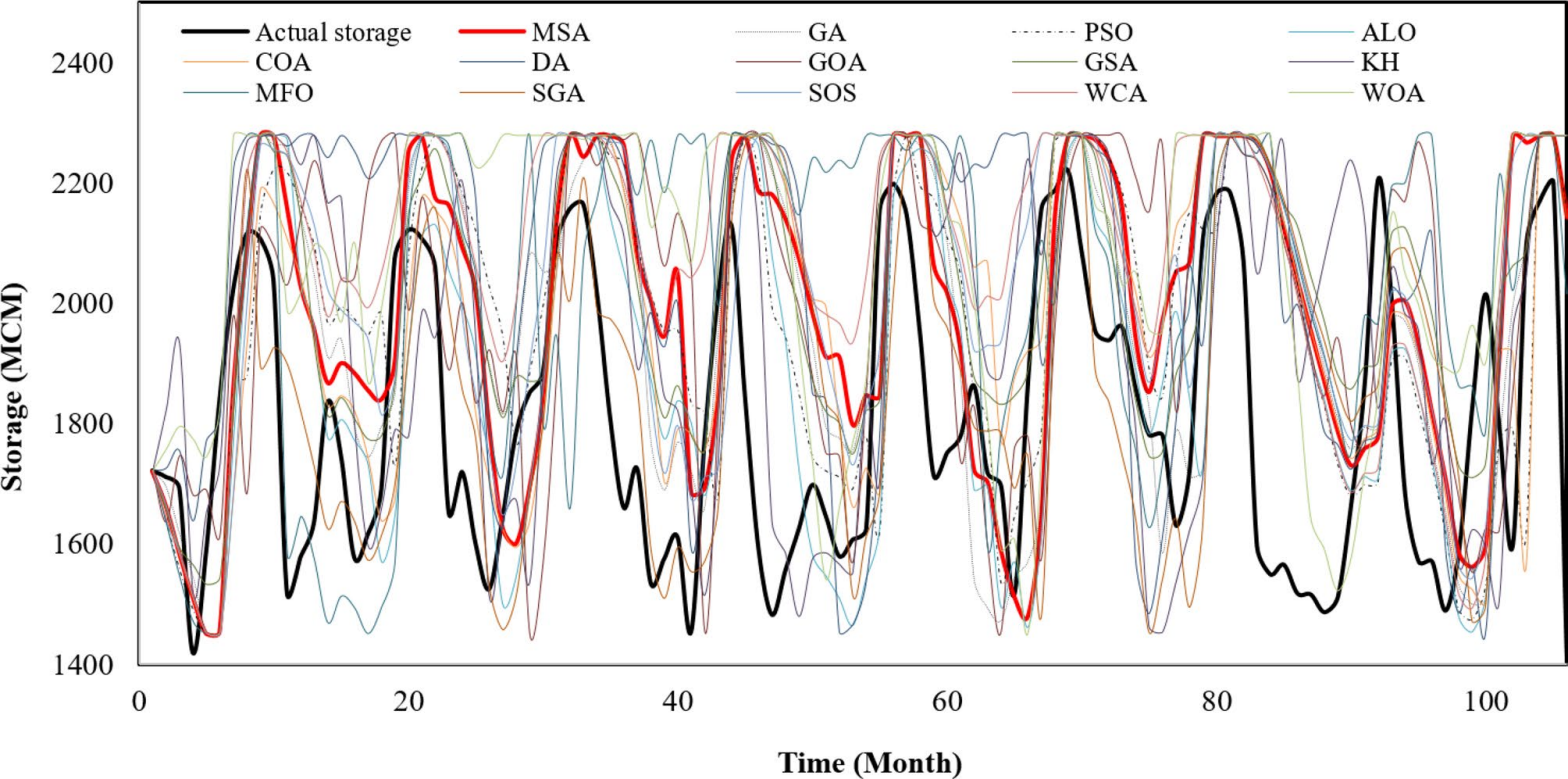
The actual storage volume of Karun-4 hydropower reservoir during operational period vs. the predicted values by the algorithms.

Figure 5 indicates the actual storage volume of Karun-4 hydropower reservoir during 106 months of operational period versus the predicted values by the algorithms. This figure is complementary to the Fig. 4. In some periods there was sufficient water in the reservoir to produce hydropower energy but it has not been used, therefore, the generation of hydropower in real operation was significantly less than the optimized operational period.

## Conclusion

Hydropower energy has been the most important source of electricity generation in the last five years. Optimizing the operation of hydropower dams is vital to maximize the hydropower generation to cope with present and future requirements. The main challenge linked to the hydropower dam operations is that the release decisions should be made in light of the system’s physical constraints, including the stochastic nature of system parameters. In this study, 14 different evolutionary algorithms were used to optimize the hydropower dams’ operation policies. These algorithms include GA, PSO, GSA, WCA, KH, SOS, ALO, MFO, SGA, WOA, DA, GOA, MSA and COA, are among the most powerful algorithms which indicated high performance in optimization of complex engineering problems. It was found that MSA placed at the first rank and was the best model in optimization of hydropower generation from Karun-4 hydropower reservoir. It had the best value of the objective function (0.147), the least value of standard deviation (0.0029), the least value of coefficient of variations (0.0192) and the shortest CPU time (19.70 s). On the contrary, the grasshopper optimization algorithm failed to produce reasonable results, so it placed at the rank of 14 among the utilized algorithm. The values of objective function, SD, CV and CPU time for this algorithm were 26.98, 7.69, 0.227 and 258.9 s, respectively. The results showed that all the utilized EAs could significantly improve the hydropower energy generation compared to the real operation of Karun-4 reservoir. Even the weakest algorithm (GOA) was able to increase the hydropower energy generation by 23%. Each of the algorithms of MSA, SGA, SOS, COA, ALO, WCA and PSO could increase the energy generation more than 65% over the 106 months operational period. Based on the obtained results, this study recommends the utilization of robust evolutionary algorithms, particularly MSA, for optimal operation of hydropower reservoirs.
